# Natural diversity in fatty acid composition and nutritional quality of African oil palm germplasm reveals opportunities for nutritional trait breeding

**DOI:** 10.3389/fpls.2026.1899181

**Published:** 2026-07-02

**Authors:** Venkatareddy Prathap, Anitha Pedapati, Govindan Ravichandran, Sanganamoni Shivashankar, Chirag Maheshwari, Shaik Sanafrian, Kancherla Suresh

**Affiliations:** 1 ICAR-Indian Institute of Oil Palm Research, Pedavegi, Andhra Pradesh, India; 2 ICAR-Indian Agricultural Research Institute, New Delhi, India

**Keywords:** carotene content, fatty acid composition, germplasm diversity, lipid quality indices, oil palm

## Abstract

**Introduction:**

Oil palm (*Elaeis guineensis* Jacq.) is the world’s most productive oil-bearing crop; however, breeding efforts have historically emphasized yield improvement, with comparatively limited focus on oil quality traits. This study aimed to characterize natural variation in oil quality and agronomic traits among African dura oil palm germplasm and identify elite accessions for breeding programs targeting nutritionally improved palm oil.

**Methods:**

A total of 83 African dura oil palm germplasm accessions originating from Cameroon and Guinea-Bissau were evaluated for fatty acid composition, lipid quality attributes, carotene content, free fatty acid content, iodine value, and fresh fruit bunch yield. Multivariate analyses, including principal component analysis (PCA), hierarchical clustering, Multi-Trait Genotype–Ideotype Distance Index (MGIDI), multivariate analysis of variance (MANOVA), and linear discriminant analysis (LDA), were employed to assess diversity and identify superior germplasm.

**Results:**

Substantial variation was observed for key oil quality traits, with palmitic acid ranging from 29.79% to 65.70% and oleic acid from 26.86% to 60.62%. PCA and hierarchical clustering revealed that germplasm differentiation was primarily driven by variation between saturated and unsaturated fatty acid fractions. MGIDI analysis identified 17 accessions possessing desirable combinations of oil quality and agronomic traits. MANOVA and LDA further confirmed significant multivariate differentiation between the prioritized accessions and the remaining germplasm.

**Conclusions:**

The identified elite accessions represent valuable genetic resources for breeding programs aimed at improving palm oil nutritional quality while maintaining agronomic performance. The study provides a robust multi-trait framework for germplasm evaluation and supports the development of nutritionally enhanced oil palm cultivars through targeted breeding.

## 1 Introduction

Oil palm (*Elaeis guineensis* Jacq.) is the most productive commercial oil-bearing crop globally, with land-use efficiency that substantially exceeds that of other major oilseed crops such as soybean, sunflower, and rapeseed (Barcelos et al., 2015). It produces 460–1,207% more vegetable oil per hectare than rapeseed or soybean while requiring comparatively lower inputs, including fertilizer and pesticides (Sibhatu, 2023). This remarkable productivity has established oil palm as a major contributor to the global vegetable oil economy (Sibhatu, 2023). Beyond its economic significance, palm oil is valued for its oxidative stability and semi-solid physicochemical properties, which make it highly versatile for a wide range of domestic and industrial food applications (Murphy et al., 2021). In addition, crude palm oil is a naturally rich source of bioactive micronutrients, particularly provitamin A carotenoids and vitamin E tocochromanols (Monde et al., 2009).

Despite these advantages, palm oil continues to be critically discussed because of its relatively high saturated fatty acid content, particularly palmitic acid, which typically constitutes 39–46% of its fatty acid profile (Mancini et al., 2015). With increasing nutritional emphasis on moderating saturated fat intake, there is growing interest in improving the lipid composition of palm oil. However, such broad generalizations often overlook the considerable biological diversity that exists within the species (Lim et al., 2020). The substantial intraspecific variation within *E. guineensis* provides a valuable opportunity for nutritional improvement through targeted genetic selection (Barcelos et al., 2015).

This biological diversity is particularly evident in the mesocarp oil composition of geographically distinct oil palm populations (Lieb et al., 2017). African oil palm populations, in particular, represent an important reservoir of biochemical diversity, with certain genotypes exhibiting lower palmitic acid levels and higher bioactive micronutrient content compared with conventional hybrids (Barcelos et al., 2015). Molecular studies further support these phenotypic differences, revealing substantial genetic differentiation and heterogeneity among breeding resources (Gan et al., 2021). Genetic studies have also identified quantitative trait loci associated with palmitic acid, oleic acid, and iodine value (IV), indicating that these quality-related traits can be effectively targeted in breeding programs (Montoya et al., 2014).

Traditionally, oil palm breeding has largely prioritized agronomic productivity, including traits such as fresh fruit bunch (FFB) yield and bunch architecture, while oil compositional traits have received comparatively less integrated attention (Maxwell et al., 2018). Similarly, many studies have focused on individual parameters, such as total carotene content or specific fatty acid proportions, rather than evaluating the overall nutritional functionality of the oil in an integrated manner (Monde et al., 2009). Such a univariate approach may not fully capture the multidimensional nature of oil quality or provide a robust framework for selecting genotypes aligned with evolving nutritional and industrial expectations (Sitepu et al., 2024).

To address this limitation, more integrated analytical frameworks are required. The nutritional functionality of an oil is influenced by the combined interaction of multiple fatty acids rather than any single component alone (Judijanto, 2025; Chen and Liu, 2020). Nutritional indices such as the Atherogenic Index (AI) and Thrombogenic Index (TI) provide a more comprehensive means of assessing lipid nutritional quality and serve as useful tools for integrated germplasm evaluation (Chen and Liu, 2020). Interpreting the multidimensional relationships among these variables benefits from multivariate analytical approaches (Jain et al., 2026). Methods such as principal component analysis (PCA) and hierarchical cluster analysis (HCA) are widely used for resolving complex compositional datasets (Granato et al., 2018). In addition, multi-trait selection frameworks such as the Multi-Trait Genotype–Ideotype Distance Index (MGIDI) enable the simultaneous prioritization of genotypes with favorable nutritional attributes alongside agronomic performance (Sitepu et al., 2024).

This study was undertaken to address the limited availability of integrated frameworks for evaluating nutritional lipid quality in oil palm germplasm. We evaluated 83 diverse dura oil palm germplasm using fatty acid profiling, derived lipid nutritional indices, and multivariate analytical approaches to identify the major drivers of compositional nutritional variation (Granato et al., 2017). Using the MGIDI framework, we prioritized germplasm based on a predefined nutritionally favorable ideotype followed by statistical validation of group differentiation through multivariate analyses. Collectively, these findings provide a data-driven framework for germplasm prioritization and support the selection of nutritionally improved oil palm breeding resources.

## 2 Materials and methods

### 2.1 Plant material and experimental site

A total of 83 IOPPV000 series dura oil palm (*Elaeis guineensis* Jacq.) germplasm, comprising 50 accessions from Cameroon and 33 from Guinea-Bissau, were evaluated in this study. These materials were introduced in 1995 under an FAO program and established in 1998 as an *ex-situ* field gene bank at the ICAR–Indian Institute of Oil Palm Research, Pedavegi, Andhra Pradesh, India (16.80° N, 81.11° E; 85 m a.s.l.) (Murugesan et al., 2020). The experimental site receives an average annual rainfall of approximately 1026 mm, with mean temperatures ranging from 24–27 °C to 34–36 °C. Germplasm accessions were planted at a standard density of 143 palms ha^-^¹ under a triangular spacing system and maintained under uniform agronomic management, including drip irrigation and recommended NPK fertilization. Mature palms were used for biochemical evaluation of mesocarp oil quality traits and FFB yield assessment. Fresh fruit bunches were harvested at physiological maturity, and mesocarp samples were processed on the same day under standardized handling conditions to minimize post-harvest biochemical variation, particularly free fatty acid (FFA) accumulation. Because fatty acid composition and related quality traits may be influenced by genotype × environment interactions, the prioritized germplasm identified in this study reflects performance under the evaluated *ex situ* conditions, and broader validation across environments would be valuable. Each of the 83 dura oil palm germplasm collections were represented by a single mature palm maintained in the field gene bank. Fruits were pooled to obtain a composite mesocarp sample for biochemical analyses.

### 2.2 Mesocarp oil extraction

Fresh mesocarp tissues were separated from ripe fruits and oven-dried at 60–70 °C until constant weight was attained. The dried mesocarp was ground into a fine powder, and approximately 2–3 g of sample was subjected to oil extraction using a Soxtherm extraction system (Gerhardt GmbH & Co. KG, Germany) with n-hexane as the extraction solvent. The extraction program consisted of hot extraction, solvent recovery, and rinsing phases according to the manufacturer’s protocol. Following extraction, residual solvent was removed by oven drying at 100–105 °C, and the extracted oil was cooled in a desiccator and stored at −20 °C until further analyses.

### 2.3 Determination of fatty acid composition by GC-FID

For each germplasm collection, fruits harvested from the representative palm were pooled to obtain a composite mesocarp sample. Fatty acid profiling was performed by GC-FID, and each sample was analyzed using three technical injection replicates. Mean values from the technical replicates were used for subsequent analyses. Fatty acids were converted to fatty acid methyl esters (FAMEs) through transesterification using 0.5 N methanolic NaOH, followed by 14% BF_3_ at 85 °C for 30 min. The resulting FAMEs were extracted with n-hexane, dried over anhydrous Na_2_SO_4_, and analyzed using a PerkinElmer Clarus 690 gas chromatograph (PerkinElmer Inc., Shelton, CT, USA) equipped with a flame ionization detector (FID) and a COL-Elite 563 capillary column (100 m × 250 µm × 0.2 µm), following our previously standardized protocol (Prathap et al., 2025a). Nitrogen was used as the carrier gas at a constant flow rate of 1.0 mL min^-^¹. The oven temperature was programmed from 150 °C (1 min hold) to 230 °C at 5 °C min^-^¹, followed by a final hold at 250 °C for 10 min. Injector and detector temperatures were maintained at 230 °C and 250 °C, respectively. Fatty acids were identified by comparison of retention times with certified FAME standards (Sigma-Aldrich) and expressed as the percentage contribution of each fatty acid to total detected fatty acids.

### 2.4 Determination of nutrition quality indices

To evaluate the lipid nutritional quality of mesocarp oil, four indices were calculated based on fatty acid composition. The Atherogenic Index (AI), Thrombogenic Index (TI), hypocholesterolemia/hypercholesterolemic ratio (H/H), and Health-Promoting Index (HPI) were calculated according to previously reported equations (Prathap et al., 2025a, b). Lower AI and TI values, together with higher H/H and HPI values, indicate a more favorable lipid nutritional profile. In the present study, lauric acid (C12:0), myristic acid (C14:0), palmitic acid (C16:0), stearic acid (C18:0), oleic acid (C18:1), and linoleic acid (C18:2) were quantified and used for the calculation of lipid nutritional quality indices.

AI = (C12:0 + 4 × C14:0 + C16:0)/(ΣMUFA + Σω-6 + Σω-3).

TI = (C14:0 + C16:0 + C18:0)/(0.5 × ΣMUFA + 0.5 × Σω-6 +  Σω-3).

H/H = (C18:1 + C18:2 + C18:3 + C18:4 + C20:4)/(C14:0  + C16:0).

HPI = (ΣMUFA + ΣPUFA)/(C12:0 + C14:0 + C16:0).

ΣMUFA primarily represented oleic acid (C18:1), whereas ΣPUFA and Σω-6 were represented by linoleic acid (C18:2). Fatty acids such as C18:3, C18:4, and C20:4 were not detected or occurred at negligible levels and therefore did not contribute meaningfully to the calculated indices.

### 2.5 Determination of free fatty acid content

Free fatty acid (FFA) content was determined using a previously reported standard titrimetric method (Canelli et al., 2020). Approximately 2.0 g of oil was dissolved in 50 mL of pre-neutralized isopropyl alcohol and titrated against 0.1 N NaOH using phenolphthalein as the indicator. FFA content was expressed as percentage palmitic acid equivalents (molecular weight = 256.4 g mol^-^¹) using the following equation:


FFA (%)=(V×N×25.6)/m.

where V represents the volume of NaOH used (mL), N is the normality of NaOH, m is the sample mass (g), and 25.6 is the conversion factor for palmitic acid.

### 2.6 Determination of iodine value

The IV, an estimate of the overall degree of lipid unsaturation, was calculated from the fatty acid composition data generated by GC-FID rather than determined experimentally. IV was expressed as the grams of iodine (I_2_) theoretically absorbed per 100 g of oil according to the previously reported equation (Kyriakidis and Katsiloulis, 2000).


IV=(0.95 × % palmitoleic acid)+(0.86 × % oleic acid)+(1.732 × % linoleic acid)+(2.616 × % linolenic acid).

### 2.7 Determination of total carotene content

Total carotene content was determined using a spectrophotometric method and expressed as β-carotene equivalents (Nawrocka and Lamorsk, 2013). Oil samples (5 g) were dissolved in 100 mL cyclohexane, and absorbance was measured at 445 nm using a GENESYS™ 180 UV–visible spectrophotometer (Thermo Scientific™, USA), with cyclohexane used as the blank. Total carotene content was expressed as mg β-carotene equivalents per kg of oil using the following equation:


Carotene(mg/kg)=[(As−Ab)×383]/W.

where As is the sample absorbance, Ab is the blank absorbance, and W is the sample weight (g). We have also clarified that the constant 383 is derived from the specific extinction coefficient of β-carotene in cyclohexane and the dilution factor used in the assay.

### 2.8 Determination of fresh fruit bunch yield

The FFB yield data used in this study were recorded during 2011, 2012, and 2013 to account for year-to-year variation in productivity. Bunches were harvested at physiological maturity, defined by the detachment of at least two loose fruits per bunch. For each palm, bunch number and bunch weight were recorded at each harvest using a calibrated digital balance. FFB yield was expressed as kg palm^-^¹ year^-^¹, calculated from cumulative bunch production over the evaluation period according to previously described methodology (Corley, 2018).

### 2.9 Statistical analysis

All statistical analyses were performed in R (version 4.4.1). Quantitative variables were standardized using Z-score normalization before multivariate analyses to ensure comparability among traits measured on different scales. Associations among fatty acid composition, lipid nutritional quality indices, physicochemical traits, carotene content, and FFB yield were evaluated using Spearman’s rank correlation analysis, with significance assessed at p < 0.05. To resolve multidimensional trait variation, principal component analysis (PCA) was performed using standardized variables. Hierarchical cluster analysis (HCA) was conducted using a correlation-based distance matrix with Ward’s minimum variance method (Ward.D2). Cluster robustness was evaluated using average silhouette width, Calinski–Harabasz index, and Gap statistic. Integrated multi-trait germplasm prioritization was performed using the Multi-Trait Genotype–Ideotype Distance Index (MGIDI) implemented in the metan package. To avoid redundancy arising from the inclusion of both primary fatty acids and nutritional quality indices derived from the same fatty acids, the MGIDI analysis was conducted using a non-redundant trait set comprising myristic acid, palmitic acid, stearic acid, oleic acid, linoleic acid, free fatty acid content, carotene content, and FFB yield. The ideotype was defined to represent germplasm combining improved nutritional quality and agronomic performance. Myristic acid, palmitic acid, stearic acid, and free fatty acid content were designated for minimization because higher concentrations are generally considered less desirable from nutritional and oil quality perspectives. Oleic acid, linoleic acid, carotene content, and FFB yield were designated for maximization because of their favorable contributions to nutritional value, provitamin A content, and productivity. No explicit weighting scheme was imposed during MGIDI analysis; instead, trait contributions were determined by the multivariate factor structure of the dataset following factor analysis. A selection intensity of 20% was applied to identify germplasm with the closest proximity to the predefined ideotype. To characterize differences between prioritized and remaining germplasm, multivariate analysis of variance (MANOVA) followed by linear discriminant analysis (LDA) was performed. Normality was assessed using the Shapiro–Wilk test, and trait-wise comparisons between prioritized and remaining germplasm groups were conducted using the Wilcoxon rank-sum test. Graphical visualizations were generated using relevant R packages.

## 3 Results and discussion

### 3.1 Natural variation in fatty acid composition, nutritional lipid quality, biochemical and yield traits across oil palm germplasm

#### 3.1.1 Fatty acid composition and profile diversity across oil palm germplasm

**Figure 1:**
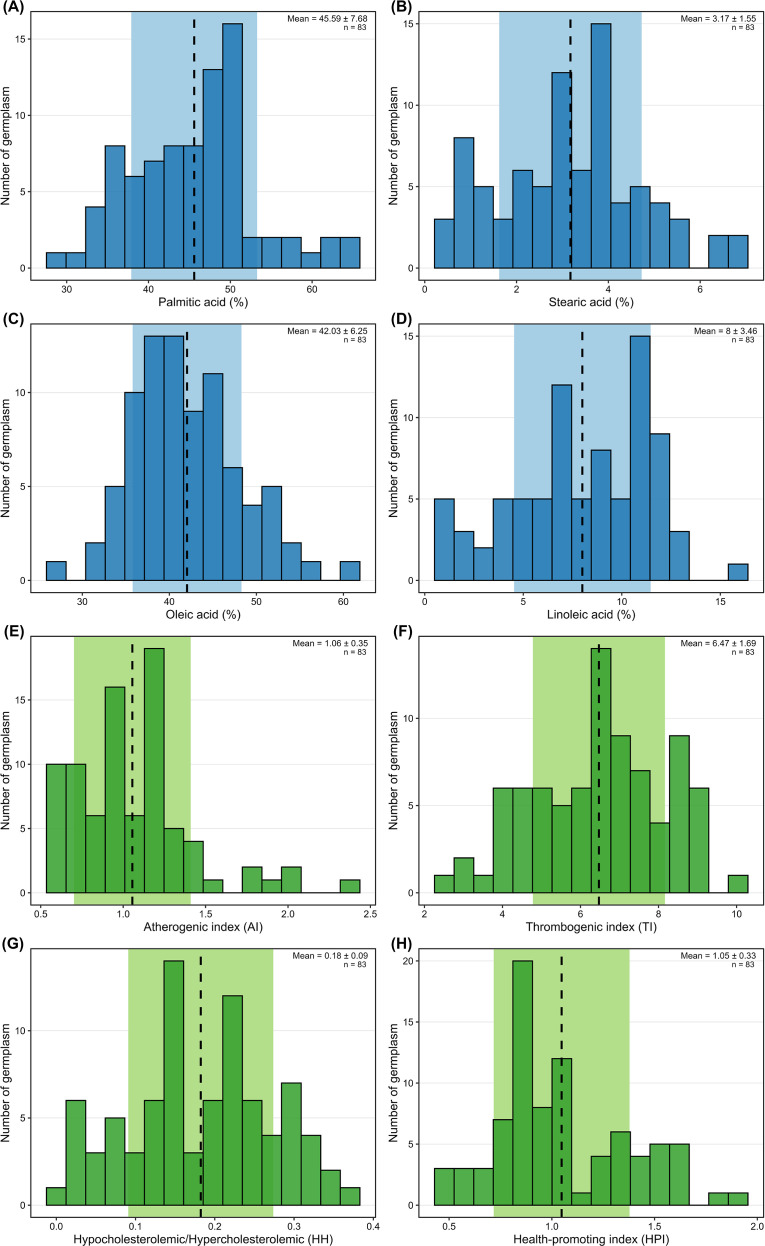
Distribution of fatty acid composition and derived nutritional lipid quality indices. Frequency distributions of major mesocarp oil fatty acids and derived lipid nutritional indices among evaluated germplasm accessions: **(A)** palmitic acid (C16:0), **(B)** stearic acid (C18:0), **(C)** oleic acid (C18:1), **(D)** linoleic acid (C18:2), **(E)** atherogenic index (AI), **(F)** thrombogenic index (TI), **(G)** hypocholesterolemic/hypercholesterolemic ratio (HH), and **(H)** health-promoting index (HPI). Dashed vertical lines indicate mean values, and shaded regions represent ±1 standard deviation.

The evaluated African dura oil palm germplasm exhibited marked diversity in mesocarp oil fatty acid composition, indicating extensive biochemical variation in lipid profiles (**Figures 1A–D**). Palmitic acid, the predominant saturated fatty acid, ranged from 29.79% to 65.70%, with a mean of 45.59 ± 7.68% (**Figure 1A**). While most germplasms were distributed within the conventional commercial palm oil range (40–55%), several germplasms exhibited markedly lower palmitic acid levels. The lowest values were recorded in IOPPV000494 (29.79%), IOPPV000023 (30.48%), IOPPV000155 (33.69%), and IOPPV000160 (34.13%). Oleic acid, the principal monounsaturated fatty acid, also showed wide variation, ranging from 26.86% to 60.62%, with a mean of 42.03 ± 6.25% (**Figure 1C**). Most germplasm clustered between 38% and 48%, whereas a subset exhibited pronounced oleic acid enrichment. The highest oleic acid levels were observed in IOPPV000023 (60.62%), followed by IOPPV000126 (55.69%), IOPPV000453 (54.00%), and IOPPV000494 (53.39%). Stearic acid ranged from 0.33% to 6.73%, averaging 3.17 ± 1.55% (**Figure 1B**), whereas linoleic acid varied from 0.61% to 15.52%, with a mean of 8.00 ± 3.46% (**Figure 1D**). Germplasm with elevated linoleic acid included IOPPV000161 (15.52%), IOPPV000133 (13.22%), and IOPPV000211 (12.98%). Myristic acid, a minor saturated fatty acid, ranged from 0.12% to 2.99%. A clear reciprocal distribution between palmitic acid and oleic acid was evident across the germplasm, with germplasm exhibiting reduced palmitic acid generally showing elevated oleic acid levels. The observed palmitic acid range exceeded values commonly reported for commercial oil palm populations, highlighting the broader compositional diversity present within this African dura germplasm. This variability provides a strong basis for germplasm prioritization targeting nutritionally desirable fatty acid profiles.

The wide palmitic acid range (29.79–65.70%) substantially exceeds the narrower ranges typically reported for commercial oil palm populations, which generally contain approximately 44% palmitic acid and 40% oleic acid (España et al., 2018; Montoya et al., 2014). Similarly, genome-wide association studies in oil palm have reported comparatively narrower variation for palmitic acid content, reinforcing the broader compositional diversity captured within the present germplasm set (Xia et al., 2019). Such expanded variability is particularly relevant because fatty acid composition remains one of the most critical determinants of both nutritional quality and technological functionality in palm oil (Barcelos et al., 2015). A pronounced reciprocal relationship between palmitic acid and oleic acid was evident across the germplasm, with reduced saturated fatty acid accumulation generally associated with increased monounsaturated fatty acid content (Montoya et al., 2014).

This inverse pattern is well documented in oil palm and reflects the coordinated regulation of fatty acid biosynthesis, where shifts in carbon partitioning influence the balance between saturated and unsaturated lipid fractions (Li et al., 2022). The observed variation is likely governed by differential regulation of key enzymes involved in fatty acid elongation, chain termination, and desaturation, including β-ketoacyl-ACP synthase II, palmitoyl-ACP thioesters, and stearoyl-ACP desaturase (Sabbadini et al., 2021). Among these, stearoyl-ACP desaturase plays a central role in converting saturated intermediates into oleic acid, thereby influencing the final unsaturation profile of mesocarp oil (Krutkaew et al., 2013). Previous developmental studies have also shown that the temporal expression of these lipid biosynthetic enzymes during fruit maturation contributes significantly to the establishment of final oil composition (Krutkaew et al., 2013).

From a nutritional perspective, the identification of phenotypes such as IOPPV000494, characterized by reduced palmitic acid and elevated oleic acid content, is particularly noteworthy. Although palm oil is frequently discussed in relation to its relatively high saturated fatty acid content (Barcelos et al., 2015), its nutritional implications are also influenced by triacylglycerol stereospecific structure, where unsaturated fatty acids such as oleic and linoleic acid are commonly enriched at the sn-2 position, potentially influencing digestion, absorption, and metabolic handling in a manner comparable to monounsaturated lipids (Lucci et al., 2015). Reduction in myristic and palmitic acid content remains a relevant compositional objective due to their recognized hypercholesterolemic potential (Montoya et al., 2014), and the occurrence of naturally low-saturated phenotypes within the present germplasm suggests that such compositional improvement may be achievable through targeted germplasm selection (Singh et al., 2009). Previous studies have identified quantitative trait loci associated with fatty acid composition, suggesting that these traits may be amenable to genetic improvement through breeding (Montoya et al., 2014). These findings demonstrate that the evaluated germplasm harbors sufficient biochemical diversity to support the development of palm oil genotypes with more favorable fatty acid compositions.

#### 3.1.2 Nutritional lipid quality diversity across oil palm germplasm

Derived lipid nutritional quality indices revealed marked variation in the nutritional functionality of mesocarp oil across the evaluated germplasm (**Figures 1E–H**). The AI ranged from 0.54 to 2.32, with a mean of 1.06 ± 0.35, with most germplasm distributed between 0.75 and 1.25 (**Figure 1E**). The lowest AI values were recorded in IOPPV000494 (0.54) and IOPPV000023 (0.54), followed by IOPPV000155 (0.61), IOPPV000072 (0.61), and IOPPV000127 (0.61). The TI also exhibited wide variation, ranging from 2.45 to 9.98, with a mean of 6.47 ± 1.69 (**Figure 1F**). The most favorable TI values were observed in IOPPV000459 (2.45), IOPPV000453 (2.90), IOPPV000402 (3.12), IOPPV000384 (3.60), and IOPPV000023 (3.95). Indices reflecting favorable lipid nutritional characteristics showed similarly broad diversity. The H/H ratio ranged from 0.01 to 0.38, averaging 0.18 ± 0.09 (**Figure 1G**), whereas the HPI varied from 0.43 to 1.86, with a mean of 1.05 ± 0.33 (**Figure 1H**). The highest H/H values were recorded in IOPPV000161 (0.38), IOPPV000133 (0.35), IOPPV000494 (0.35), IOPPV000155 (0.32), and IOPPV000129 (0.32). Similarly, the highest HPI values were observed in IOPPV000494 (1.86), IOPPV000023 (1.85), IOPPV000155 (1.65), IOPPV000072 (1.64), and IOPPV000127 (1.63). Notably, IOPPV000494 and IOPPV000023 consistently ranked among the highest-performing germplasm across multiple lipid nutritional indices, reflecting favorable integrated fatty acid-derived nutritional profiles.

The observed diversity in lipid nutritional quality indices highlights the wide compositional variability present within the evaluated germplasm and its potential implications for nutritional functionality. The AI and TI are widely used predictive indicators for assessing the relative balance between pro-atherogenic saturated fatty acids and protective unsaturated fatty acids, with lower values generally considered more favorable from a cardiovascular nutrition perspective (Motamedzadegan et al., 2020). Elevated values of these indices have been associated with less desirable lipid profiles in dietary fat assessment (Tilami and Kouřimská, 2022). Compared with conventional *E. guineensis* palm oil, the identification of germplasm exhibiting comparatively lower AI and TI values indicates the presence of naturally occurring compositional diversity that may be useful for improving lipid nutritional quality through targeted germplasm selection (Santos et al., 2024). Lower AI and TI values generally reflect a greater proportional contribution of anti-atherogenic unsaturated fatty acids (Garrido et al., 2019).

Similarly, the H/H ratio provides an integrative measure of the relative balance between cholesterol-lowering and cholesterol-raising fatty acids, with higher values indicating a more nutritionally favorable fatty acid composition (Munhoz et al., 2018). Germplasm exhibiting higher H/H values were generally characterized by elevated unsaturated fatty acid proportions, consistent with current nutritional emphasis on replacing saturated fatty acids with monounsaturated and polyunsaturated alternatives in dietary fat quality assessment (Urugo et al., 2021). However, the metabolic effects of palm oil are not determined solely by overall fatty acid composition, as triacylglycerol stereospecific structure also influences digestion and lipid metabolism, particularly through the preferential localization of unsaturated fatty acids at the sn-2 position (Elson and Alfin-Slater, 1992).

Furthermore, the broader genus-level comparisons suggest that optimizing these indices, mirroring patterns, is a viable strategy to enhance the overall health-promoting potential of the oil profile (Kavoosi et al., 2025). This approach is particularly critical as oxidative stability must be maintained during breeding efforts, given that higher levels of unsaturated fatty acids can alter the thermo-oxidative performance of the oil (Memon et al., 2023). Consequently, evaluating the balance between polyunsaturated fatty acids and saturated fatty acids remains essential, as this ratio serves as a primary metric for defining the health-promoting potential of vegetable oils (Chen and Liu, 2020). Integrating such comprehensive nutritional assessments into breeding programs enables the systematic selection of genotypes that reconcile improved human health metrics with the long-term industrial requirement for oxidative stability (Ong and Goh, 2002). The significant variation in nutritional indices demonstrates that this germplasm contains rare phenotypes with lipid profiles that are naturally optimized for human health. These findings provide a robust foundation for breeding programs aimed at developing palm oil hybrids that offer both high stability and a superior cardiovascular safety profile.

#### 3.1.3 Free fatty acid diversity across oil palm germplasm

**Figure 2:**
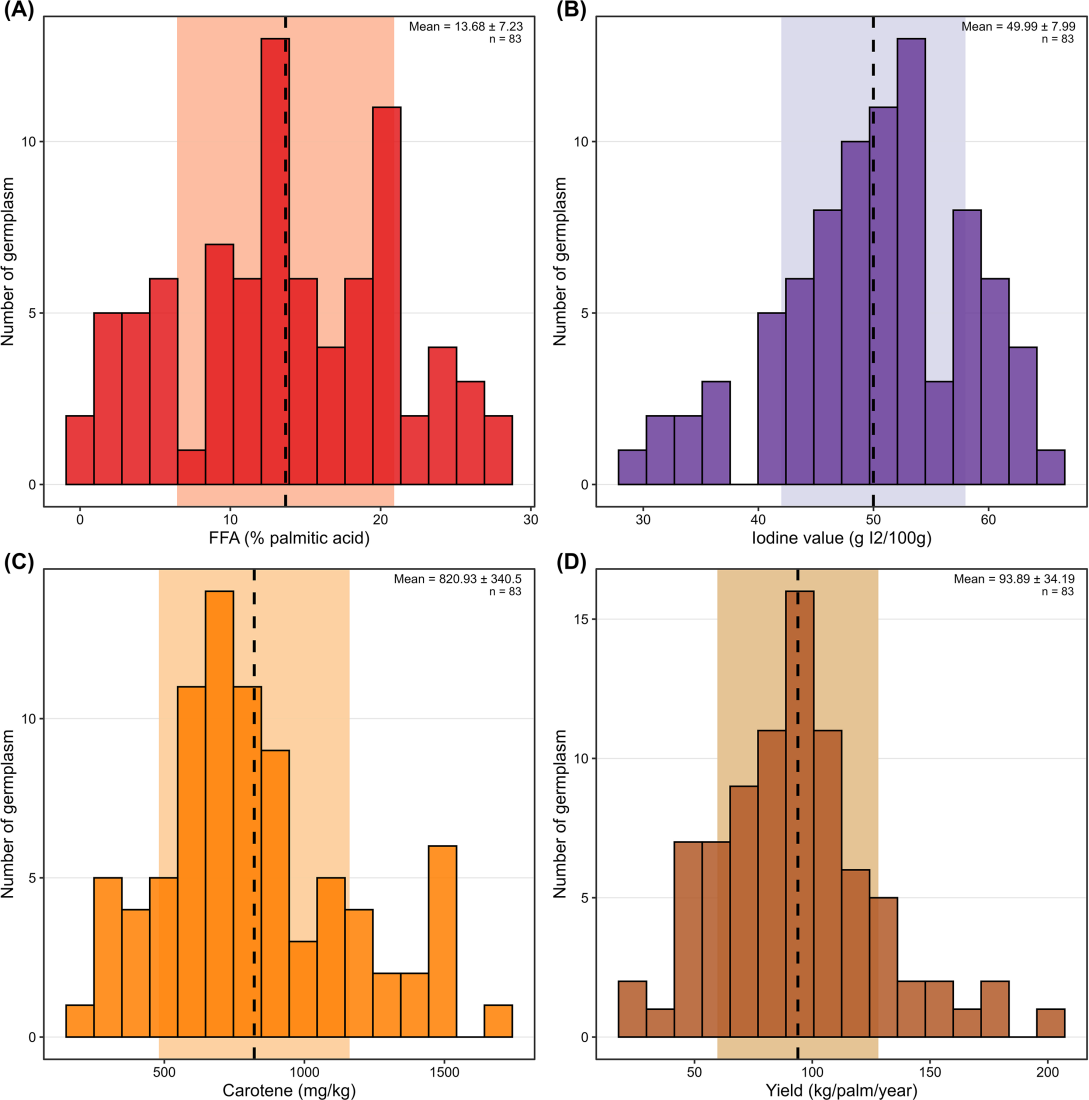
Distribution of hydrolytic quality, lipid unsaturation, carotene content, and fresh fruit bunch yield. Frequency distributions of oil quality and agronomic traits: **(A)** free fatty acid (FFA) content (% palmitic acid basis), **(B)** iodine value (g I_2_/100 g oil), **(C)** total carotene concentration (mg kg^-^¹), and **(D)** fresh fruit bunch (FFB) yield (kg palm^-^¹ year^-^¹). Dashed vertical lines indicate mean values, and shaded regions represent ±1 standard deviation.

The FFA content, a widely used indicator of hydrolytic oil quality, exhibited pronounced variation across the evaluated African dura oil palm germplasm (**Figure 2A**). FFA values, expressed as palmitic acid equivalents, ranged from 0.59% to 28.43%, with an overall mean of 13.68 ± 7.23%. Most germplasm was distributed within the 8–20% range, although a subset exhibited markedly lower FFA accumulation. The lowest FFA levels were recorded in IOPPV000113 (0.59%), IOPPV000015 (0.80%), IOPPV000234 (1.24%), IOPPV000002 (1.64%), and IOPPV000023 (1.80%). In contrast, several germplasms exhibited substantially elevated FFA levels, approaching 28%, indicating wide diversity in hydrolytic stability across the germplasm.

The wide variation in FFA levels observed across the evaluated germplasm indicates substantial diversity in hydrolytic oil stability, an important determinant of crude palm oil quality. In oil palm, FFA accumulation primarily results from the hydrolysis of triacylglycerols by endogenous lipase activity during fruit ripening or following tissue disruption (Wong et al., 2015). Elevated FFA levels negatively affect crude oil quality and increase refining requirements, making low-FFA germplasm particularly desirable for edible oil production (Barcelos et al., 2015). The marked contrast between high-FFA germplasm and low-acidity phenotypes such as IOPPV000113 highlights considerable variation in lipase-mediated hydrolysis among the evaluated materials (Ebongue et al., 2014). Previous studies have reported that high acidity is generally associated with the dominant allele Pa, whereas low acidity is linked to the recessive allele pa, providing a biological basis for the inheritance of low-lipase phenotypes (Benoit et al., 2016). Furthermore, certain low-lipase genotypes have been shown to maintain negligible FFA accumulation even under conditions that normally promote lipolysis (Ebongue et al., 2008). Molecular studies have also identified genomic regions associated with reduced post-harvest oil deterioration, and marker systems such as mEgCIR_LIP03 offer opportunities for early-stage selection of favorable materials (Morcillo et al., 2013; Weng, 2025). Collectively, these findings highlight the value of low-FFA germplasm for improving crude palm oil quality, post-harvest stability, and processing efficiency (Barcelos et al., 2015).

#### 3.1.4 Iodine value diversity across oil palm germplasm

Mesocarp oil IV, reflecting overall lipid unsaturation, exhibited marked variation across the evaluated African dura oil palm germplasm (**Figure 2B**). IV ranged from 28.15 to 64.17 g I_2_/100 g oil, with an overall mean of 49.99 ± 7.99 g I_2_/100 g. Most germplasm were distributed within the 45–55 g I_2_/100 g interval, although a subset exhibited comparatively higher IV, indicating greater unsaturation. The highest IV values were recorded in IOPPV000023 (64.17 g I_2_/100 g), IOPPV000494 (62.55 g I_2_/100 g), IOPPV000126 (59.69 g I_2_/100 g), IOPPV000453 (58.21 g I_2_/100 g), and IOPPV000388 (57.83 g I_2_/100 g). These high-IV germplasm generally corresponded to lower palmitic acid and elevated oleic acid content, whereas germplasm with lower IV were associated with comparatively more saturated fatty acid profiles.

The broad range of IV observed (28.15–64.17 g I_2_/100 g) reflects substantial variation in the overall degree of oil unsaturation among the evaluated germplasm, an important characteristic influencing the physicochemical properties and end-use functionality of palm oil. Conventional E. guineensis typically exhibits moderate IV, whereas E. oleifera and interspecific hybrids often show higher values because of their greater unsaturated fatty acid content (Singh et al., 2009). The identification of dura germplasm with IV exceeding 57 g I_2_/100 g, such as IOPPV000023, indicates the presence of accessions possessing comparatively more unsaturated lipid profiles approaching the upper range reported for conventional E. guineensis materials (Montoya et al., 2014). This variation is consistent with the inverse relationship between saturated fatty acid accumulation, particularly palmitic acid, and overall lipid unsaturation, whereby reduced saturated fatty acid content contributes to elevated IV (Sujadi et al., 2018).

Previous studies have shown that variation in IV is associated with multiple biological processes involved in fatty acid biosynthesis and desaturation (Mozzon et al., 2018). Quantitative trait loci associated with IV and related fatty acid traits have been reported across several genomic regions, including linkage Group 1 and regions OT1, T2, T3, and T9 (Singh et al., 2009). Furthermore, moderate to high heritability has been reported for several compositional traits related to oil unsaturation, indicating potential opportunities for improvement through breeding (Singh et al., 2009). The occurrence of naturally high-IV phenotypes within pure E. guineensis germplasm broadens the available breeding resources for improving oil unsaturation and enhancing the nutritional and functional quality of palm oil (Montoya et al., 2014).

#### 3.1.5 Carotene diversity across oil palm germplasm

Total carotene concentration exhibited marked variation across the evaluated African dura oil palm germplasm, indicating broad diversity in mesocarp carotenoid accumulation (**Figure 2C**). Total carotene content ranged from 248.33 to 1769.41 mg kg^-^¹, with an overall mean of 820.93 ± 340.5 mg kg^-^¹. Most germplasm was distributed within the 500–1000 mg kg^-^¹ interval, although a subset exhibited notably elevated carotene accumulation. The highest carotene concentrations were recorded in IOPPV000079 (1769.41 mg kg^-^¹), followed by IOPPV000494 (1567.32 mg kg^-^¹), IOPPV000129 (1518.86 mg kg^-^¹), IOPPV000161 (1492.74 mg kg^-^¹), and IOPPV000023 (1438.56 mg kg^-^¹). In contrast, IOPPV000055 exhibited the lowest carotene concentration (248.33 mg kg^-^¹), highlighting substantial variation in carotenoid content across the germplasm panel.

The wide range in carotene content observed among the evaluated dura germplasm demonstrates substantial phenotypic variation in provitamin A carotenoid accumulation. Crude palm oil is recognized as one of the richest natural sources of α-carotene and β-carotene, and the high-carotene accessions identified in this study exceeded values commonly reported for conventional crude palm oil (Barcelos et al., 2015; Suraninpong and Nuanlaong, 2022). Previous studies have shown that carotene concentration varies considerably among oil palm germplasm and is influenced by both biological and environmental factors (Singh et al., 2013; Montoya et al., 2014). The identification of germplasm combining elevated carotene content with desirable compositional and agronomic characteristics highlights their potential utility in breeding programs aimed at improving the nutritional quality of palm oil while maintaining productivity (Sambanthamurthi et al., 2000; Ting et al., 2016).

#### 3.1.6 Yield diversity across oil palm germplasm

The FFB yield exhibited marked agronomic variation across the evaluated African dura oil palm germplasm (**Figure 2D**). Annual FFB yield ranged from 34.18 to 197.40 kg palm^-^¹ year^-^¹, with an overall population mean of 93.89 ± 34.19 kg palm^-^¹ year^-^¹. Most germplasm was distributed within the 60–120 kg palm^-^¹ year^-^¹ range, although a subset displayed comparatively higher productivity. The highest FFB yield was recorded in IOPPV000124 (197.40 kg palm^-^¹ year^-^¹), followed by IOPPV000310 (178.62 kg palm^-^¹ year^-^¹), IOPPV000411 (168.35 kg palm^-^¹ year^-^¹), IOPPV000161 (156.84 kg palm^-^¹ year^-^¹), and IOPPV000023 (149.72 kg palm^-^¹ year^-^¹). The lowest observed yield was 34.18 kg palm^-^¹ year^-^¹, highlighting substantial productivity variation across the germplasm panel.

The broad variation in FFB yield (34.18–197.40 kg palm^-^¹ year^-^¹) highlights the considerable agronomic diversity present within the evaluated dura germplasm. Because all germplasm was maintained under uniform agronomic management and irrigation, the observed variation likely reflects intrinsic differences among germplasm, although genotype × environment interactions remain relevant (Maxwell et al., 2018). Oil palm yield is a complex quantitative trait influenced by multiple components, including bunch number and average bunch weight, both of which have been reported to exhibit high variability and moderate to high heritability in African germplasm populations (Swaray et al., 2020).

The broad variation in FFB yield highlights the considerable agronomic diversity present within the evaluated dura germplasm. Because all germplasm was maintained under uniform agronomic management and irrigation, the observed variation likely reflects intrinsic differences among germplasm, although genotype × environment interactions remain relevant (Maxwell et al., 2018). Oil palm yield is a complex trait influenced by multiple components, including bunch number and average bunch weight, both of which have been reported to exhibit substantial variability in African germplasm populations (Swaray et al., 2020). The identification of high-yielding germplasm remains important for narrowing the gap between current plantation productivity and the potential of elite planting materials (Barcelos et al., 2015; Wahome et al., 2023).

Germplasm such as IOPPV000023 and IOPPV000161 are particularly noteworthy because they combine comparatively high FFB yield with favorable lipid nutritional characteristics. Consistent with the relatively weak correlation observed between yield and iodine value, these findings suggest that improvement of agronomic performance and oil quality may be achievable simultaneously (Singh et al., 2009). This is particularly relevant for the development of commercially valuable tenera (D × P) hybrids, where both productivity and oil quality are important breeding objectives (Arolu et al., 2016; Murugesan et al., 2020). Overall, the observed diversity confirms the value of this African dura germplasm as breeding resources for the development of high-yielding oil palm cultivars with improved nutritional attributes (Swaray et al., 2020).

### 3.2 Synergies and trade-offs: interrelationships among traits

**Figure 3:**
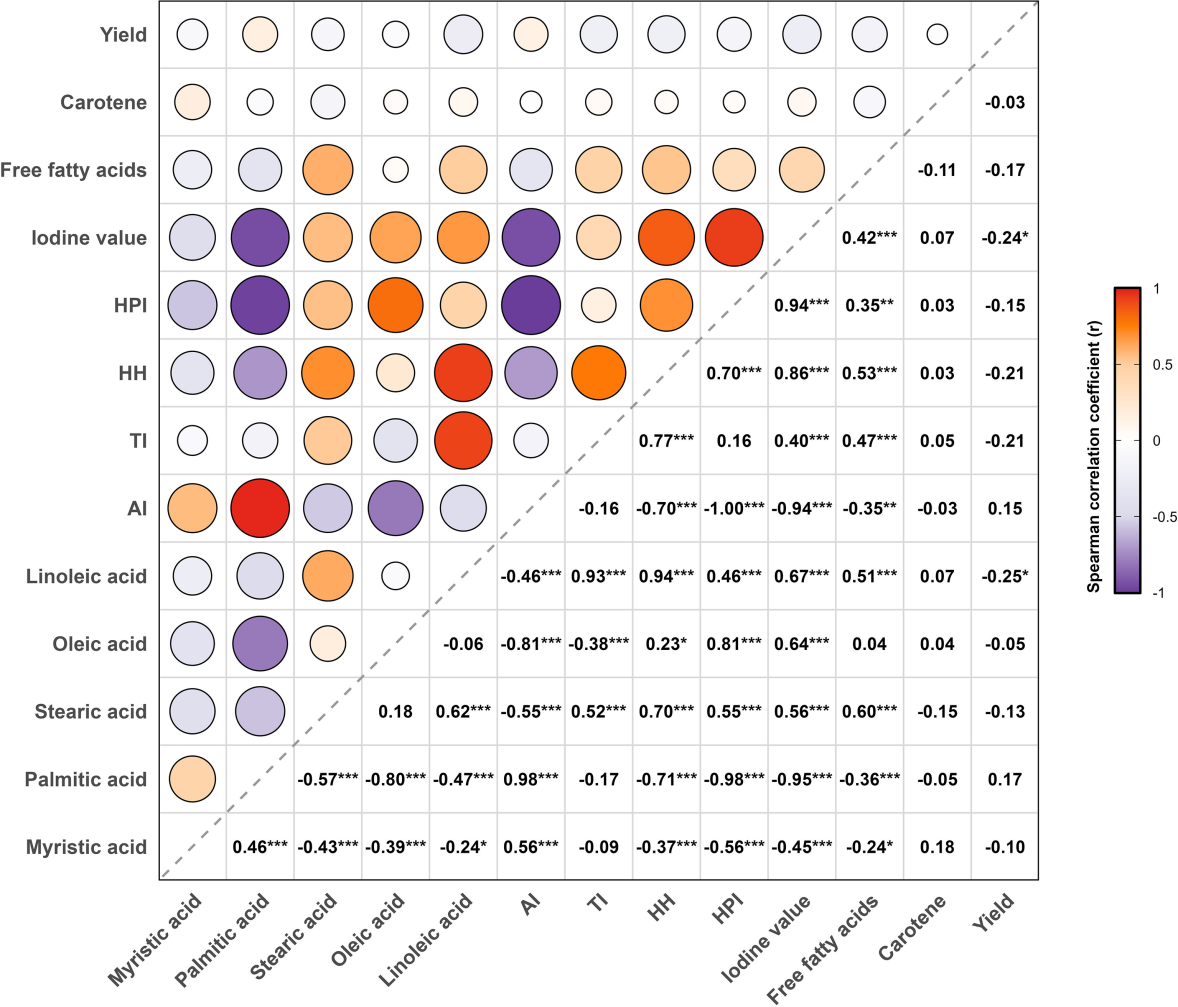
Spearman correlation matrix showing interrelationships among fatty acid composition, nutritional lipid quality indices, physicochemical traits, carotene content, and yield in oil palm germplasm. Circle size and color intensity indicate correlation strength and direction, while numerical values represent correlation coefficients. Asterisks indicate significance levels (*p < 0.05, **p < 0.01, ***p < 0.001).

Spearman correlation analysis revealed clear interrelationships among fatty acid composition, derived lipid nutritional quality indices, physicochemical traits, carotene content, and yield (**Figure 3**). The correlation structure was characterized by strong reciprocal associations between saturated and unsaturated fatty acid fractions, most notably the pronounced negative correlation between palmitic acid (C16:0) and oleic acid (C18:1) (r = −0.80, p < 0.001). Palmitic acid also showed significant negative correlations with linoleic acid (C18:2) (r = −0.47, p < 0.001) and stearic acid (C18:0) (r = −0.57, p < 0.001). Derived lipid nutritional indices exhibited strong expected relationships with their constituent fatty acid traits. The AI showed a strong positive correlation with palmitic acid (r = 0.98, p < 0.001) and a strong inverse relationship with the HPI (r = −1.00, p < 0.001), reflecting their formula-based dependence on fatty acid composition. IV showed strong positive associations with oleic acid (r = 0.64, p < 0.001), linoleic acid (r = 0.67, p < 0.001), H/H ratio, and HPI (r = 0.94, p < 0.001), while exhibiting a strong negative correlation with palmitic acid (r = −0.95, p < 0.001). Carotene content displayed generally weak correlations with most measured traits, suggesting relative independence from major fatty acid compositional variation. Yield also exhibited limited associations with the evaluated biochemical traits, with only a modest negative correlation observed with IV (r = −0.24, p < 0.05).

The strong inverse relationship between palmitic and oleic acids reflects the coordinated metabolic balance within the fatty acid biosynthetic pathway of oil palm, where carbon partitioning influences the relative accumulation of saturated and unsaturated fatty acid fractions (Singh et al., 2009). This high-magnitude negative correlation suggests that selection for reduced palmitic acid may be accompanied by increased oleic acid accumulation, an outcome of potential relevance for modifying oil compositional properties (Montoya et al., 2014). The near-perfect correlation between palmitic acid and the AI further indicates that C16:0 is a dominant contributor to this derived nutritional metric, consistent with its recognized contribution to lipid nutritional assessment frameworks (Chen and Liu, 2020). Similarly, the strong inverse relationship between AI and HPI reflects the shared dependence of these indices on underlying fatty acid composition (Chen and Liu, 2020).

IV also emerged as a useful integrative indicator of overall lipid unsaturation, as reflected by its strong associations with individual unsaturated fatty acids and derived nutritional indices such as H/H and HPI (Sujadi et al., 2018). Its strong negative relationship with palmitic acid (r = −0.95) further supports its utility as a compositional screening parameter for identifying germplasm with relatively more unsaturated lipid profiles. The weak associations observed between carotene content and major fatty acid traits suggest that carotenoid accumulation may be regulated somewhat independently of fatty acid composition (Noh et al., 2002). Likewise, the relatively weak relationship between yield and compositional quality traits, including the modest association with IV, indicates limited coupling between agronomic productivity and key biochemical quality parameters in the evaluated germplasm (Barcelos et al., 2015). These correlation patterns highlight the importance of palmitic–oleic compositional balance as a central determinant of lipid nutritional quality while suggesting that multiple desirable traits may be simultaneously considered during germplasm prioritization.

### 3.3 Multidimensional structuring via principal component analysis

**Figure 4:**
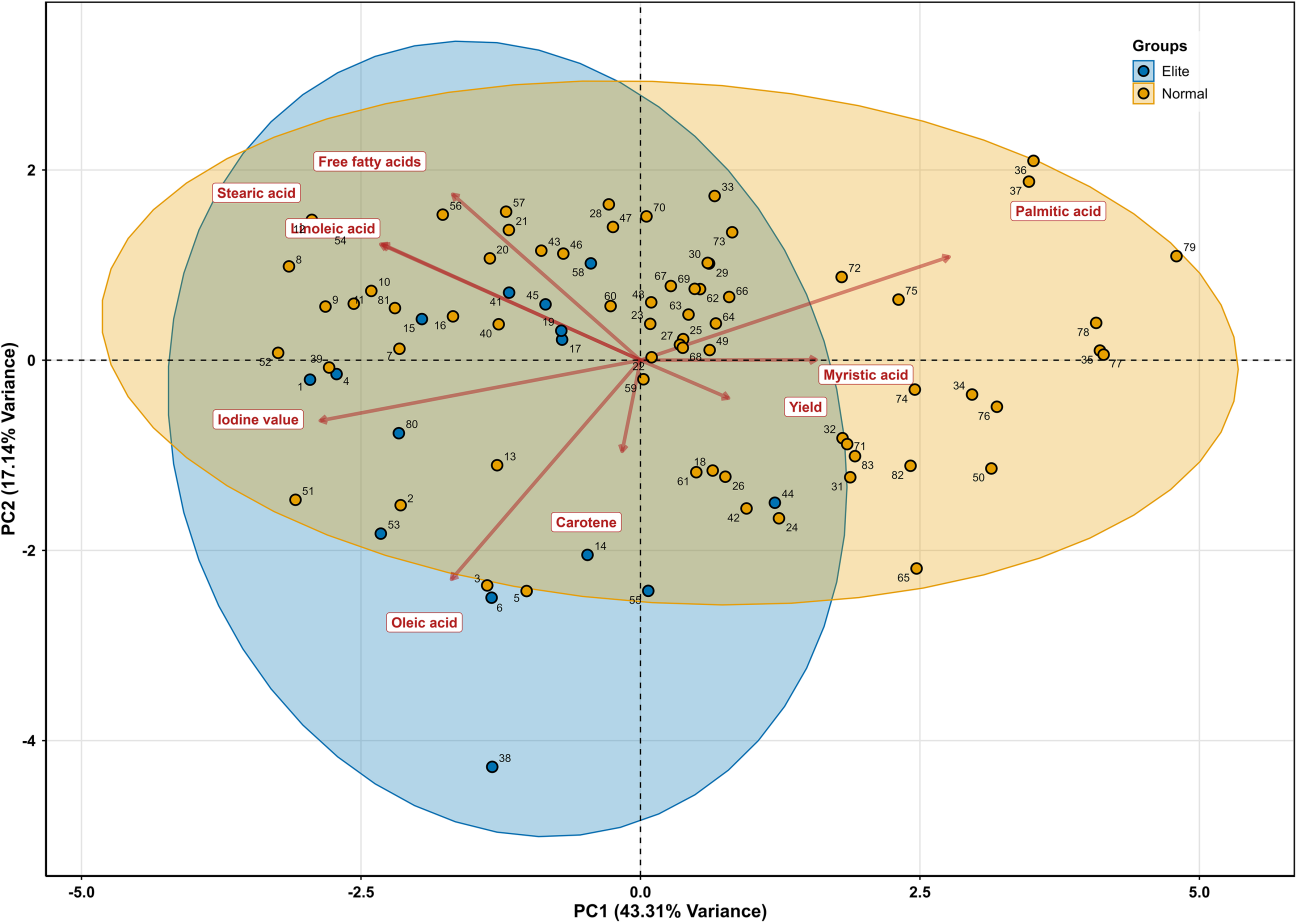
Principal component analysis (PCA) biplot illustrating the distribution of 83 African *dura* oil palm germplasm accessions and trait loadings based on fatty acid composition, nutritional lipid quality indices, physicochemical traits, carotene content, and yield. PC1 (43.31%) and PC2 (17.14%) together explained 60.45% of the total variance. Ellipses indicate dispersion of elite and normal germplasm groups.

The PCA was performed to resolve the multidimensional structure underlying variation in fatty acid composition, derived lipid nutritional quality indices, physicochemical traits, carotene content, and yield across the evaluated oil palm germplasm (**Figure 4**). The first two principal components explained a combined 60.45% of the total variance, with PC1 accounting for 43.31% and PC2 accounting for 17.14%. The first two principal components explained 60.45% of the total variance, while the first three and first four principal components accounted for 71.4% and 83.1% of the total variance, respectively. This indicates that although PC1 and PC2 captured the major patterns of variation, additional components also contributed meaningfully to the overall multivariate structure of the dataset. The distribution of trait loadings indicated that PC1 primarily captured variation associated with lipid compositional quality, separating saturated fatty acid-associated traits from unsaturation-linked traits. Palmitic acid (C16:0), myristic acid (C14:0), and the AI loaded in the opposite direction to oleic acid (C18:1), linoleic acid (C18:2), IV, the H/H ratio, and the HPI, reflecting the contrasting distribution of saturated and unsaturated lipid characteristics. In contrast, PC2 captured additional variation associated with FFA content, carotene concentration, and yield, indicating that these traits contributed independently to overall germplasm differentiation. Germplasm prioritized through multi-trait selection was generally positioned toward regions associated with favorable unsaturation-linked nutritional traits, whereas germplasm with comparatively higher saturated fatty acid signatures occupied the opposing quadrant.

The predominance of the first principal component (43.31% variance explained) indicates that variation in fatty acid composition, particularly the balance between saturated and unsaturated lipid fractions, represents a major source of phenotypic differentiation within the evaluated oil palm germplasm (Pedapati et al., 2021). The opposing placement of palmitic and myristic acids relative to oleic and linoleic acids along the PC1 axis is consistent with the coordinated metabolic balance underlying fatty acid biosynthesis, where shifts in carbon partitioning influence the relative accumulation of saturated and unsaturated fatty acids (Yaakub et al., 2020). The close alignment of derived nutritional indices such as AI and H/H with these compositional traits further supports the biological relevance of this axis in capturing lipid nutritional variation (Chen and Liu, 2020).

The co-localization of IV with unsaturation-associated nutritional indices further supports its utility as an integrative indicator of multidimensional oil compositional quality (Sujadi et al., 2018). In contrast, the broader distribution of yield, carotene content, and FFA levels along the second principal component (17.14% variance explained) suggests that these traits contribute additional and partially independent sources of variation relative to core fatty acid architecture (Barcelos et al., 2015). This separation is relevant from a breeding perspective, as it indicates the possibility of simultaneously considering oil compositional quality, carotenoid enrichment, and agronomic performance during germplasm selection rather than assuming strict trade-offs among these traits (Noh et al., 2002). Germplasm positioned toward regions associated with unsaturation-linked nutritional characteristics may therefore represent useful candidates for breeding programs targeting improved oil quality (Sujadi et al., 2018). The PCA demonstrates that a substantial proportion (60.45%) of the observed germplasm variation can be summarized by two major compositional axes, providing a useful framework for multidimensional trait prioritization. The clustering of favorable nutritional indices and IV on a single dimension provides a simplified framework for identifying elite genotypes with superior health-promoting characteristics. Because yield and carotene traits are located on separate axes of variation, there is high potential for the independent and simultaneous improvement of both oil quality and quantity.

### 3.4 Hierarchical clustering and nutritional similarity patterns

**Figure 5:**
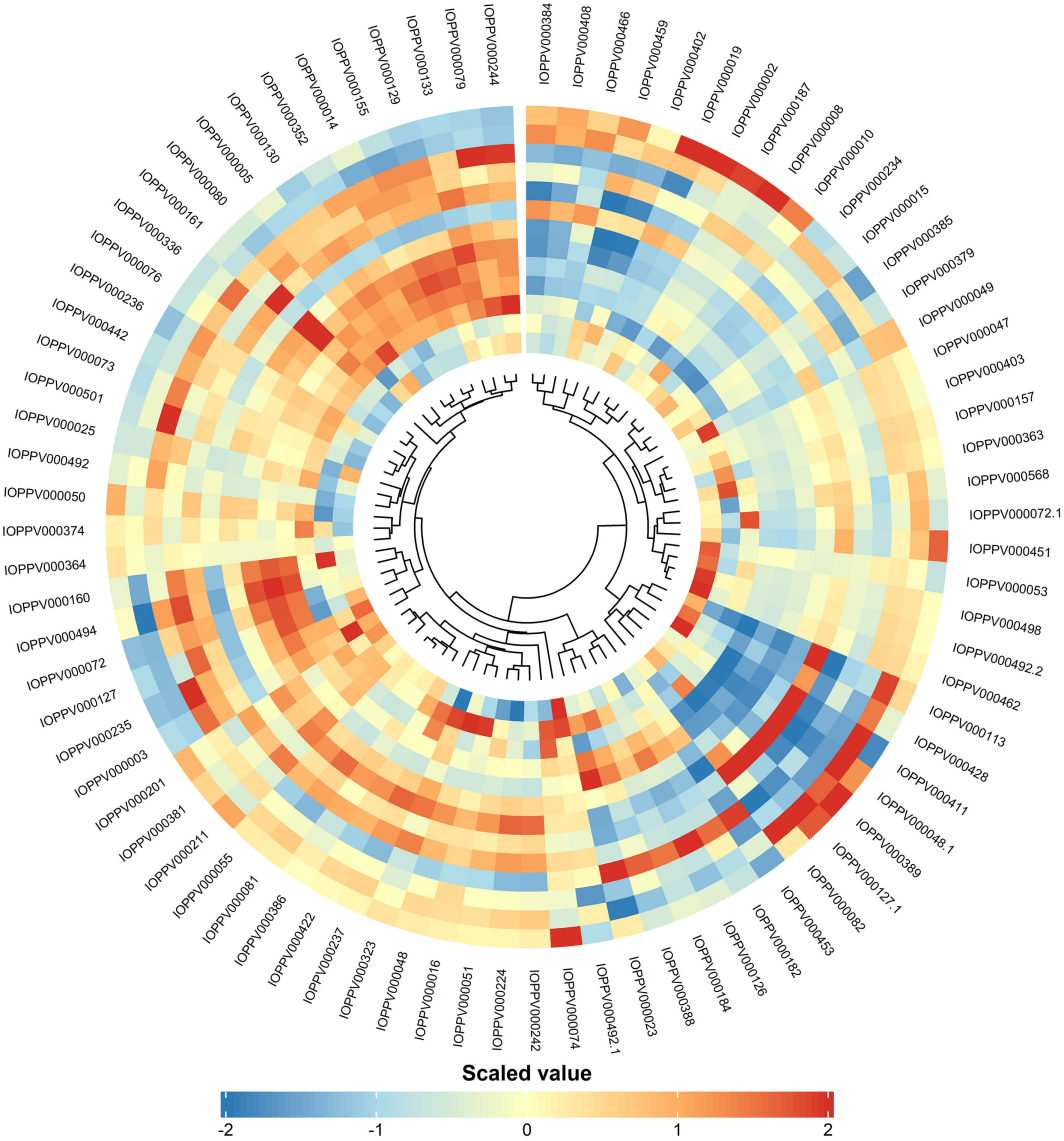
Circular hierarchical clustered heatmap showing multivariate compositional relationships among oil palm germplasm accessions. Hierarchical clustering based on standardized biochemical, nutritional, physicochemical, and agronomic traits across 83 African *dura* oil palm accessions using Ward’s clustering method. Individual accessions are arranged according to compositional similarity, and color gradients represent standardized trait values (Z-scores).

The HCA was performed to examine multivariate similarity patterns among the evaluated oil palm germplasm based on integrated fatty acid composition, derived lipid nutritional quality indices, physicochemical traits, carotene content, and yield (**Figure 5**). The clustered heatmap revealed clear trait-level organization, with saturated fatty acid-associated traits showing distinct clustering from unsaturation-linked nutritional characteristics. Palmitic acid (C16:0) and the AI clustered closely, whereas oleic acid (C18:1), linoleic acid (C18:2), IV, the H/H ratio, and the HPI formed a separate trait group associated with higher lipid unsaturation. In contrast, carotene content, FFA levels, and yield exhibited more dispersed clustering patterns across germplasm, indicating additional sources of variation beyond core fatty acid compositional structure. Germplasm-level clustering revealed substantial heterogeneity in integrated biochemical and agronomic trait profiles, with germplasm exhibiting diverse combinations of nutritional and productivity characteristics.

The application of HCA provides a useful multivariate framework for identifying trait similarity patterns and compositional groupings within the evaluated oil palm germplasm (A et al., 2015). The close clustering of palmitic acid and the AI is biologically consistent, given the dominant contribution of C16:0 to saturated lipid composition and its influence on derived nutritional indices (Chen and Liu, 2020). Likewise, the separation of saturation-associated traits from the cluster containing IV and unsaturated fatty acids reflects the broader compositional contrast between saturated and unsaturated lipid characteristics observed throughout the dataset (Yaakub et al., 2020). The co-clustering of IV with oleic and linoleic acids further supports its utility as an integrative screening parameter for identifying germplasm with relatively more unsaturated oil profiles (Sujadi et al., 2018).

The dispersed clustering behavior of carotene content, FFA levels, and yield relative to the core fatty acid trait groups suggests that these traits may be influenced by partially distinct physiological and genetic determinants (Singh et al., 2009). This interpretation is consistent with quantitative trait locus studies reporting separate genomic regions associated with fatty acid composition and other agronomic or quality traits (Ting et al., 2016). From a breeding perspective, such partial trait separation is advantageous because it suggests opportunities for simultaneous consideration of oil compositional quality, carotenoid enrichment, and agronomic performance rather than strict trait coupling (Constantin et al., 2017). The HCA highlights the compositional diversity of the evaluated germplasm and provides a useful framework for grouping germplasm according to integrated biochemical and agronomic characteristics (Pedapati et al., 2021).

### 3.5 Multi-trait chemometric prioritization of oil palm germplasm

**Figure 6:**
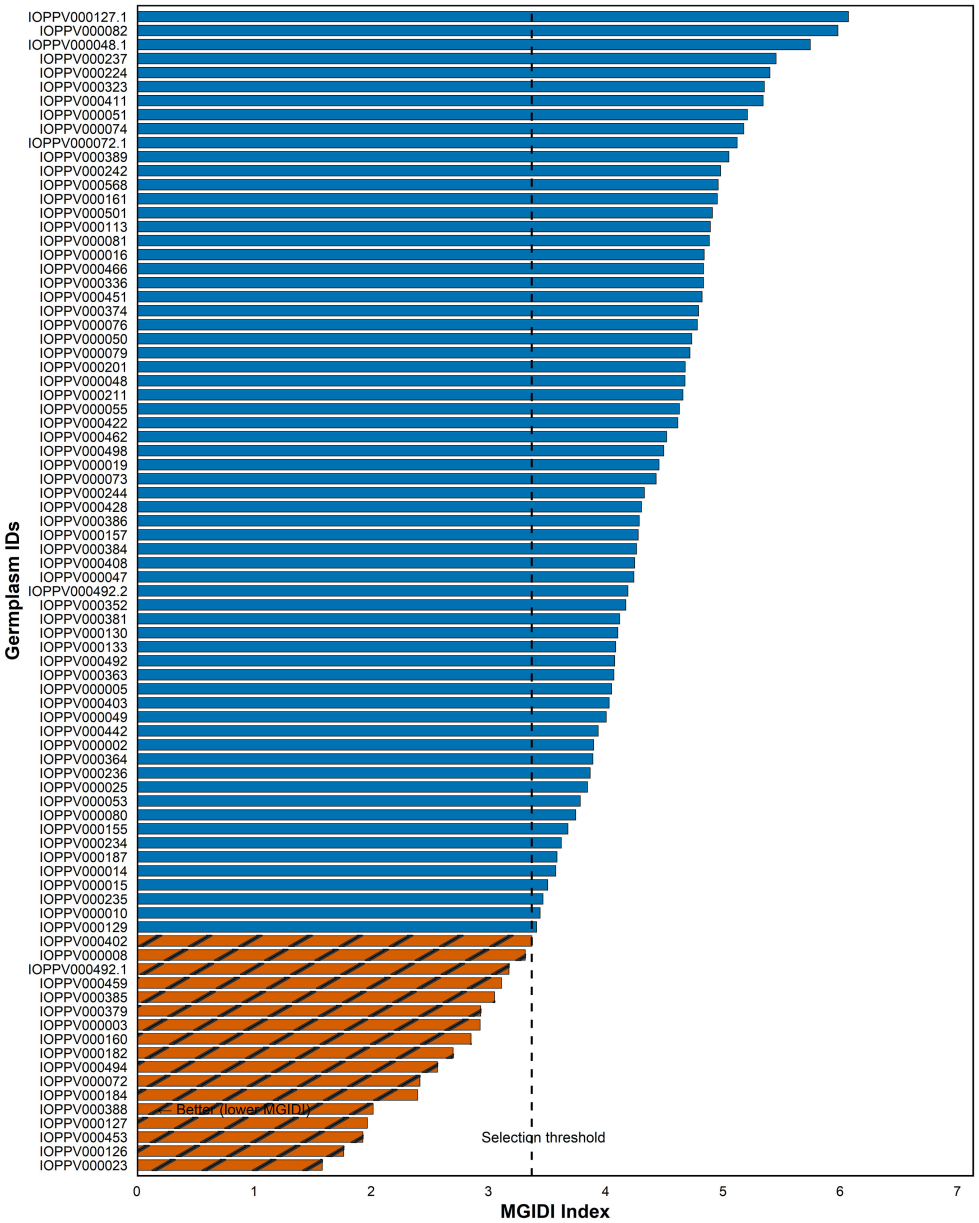
Multi-trait genotype–ideotype distance index (MGIDI) ranking of 83 African dura oil palm germplasm based on a non-redundant trait set comprising myristic acid, palmitic acid, stearic acid, oleic acid, linoleic acid, free fatty acid content, carotene content, and fresh fruit bunch yield. Lower MGIDI values indicate closer proximity to the predefined ideotype. The dashed line represents the 20% selection threshold used to identify elite germplasm.

To enable integrated multi-trait prioritization of nutritionally favorable germplasm, the MGIDI was applied by simultaneously incorporating fatty acid composition, derived lipid nutritional quality indices, physicochemical traits, carotene content, and yield (**Figure 6**). The MGIDI distribution revealed substantial heterogeneity among germplasm, with most germplasm exhibiting moderate-to-high index values, whereas a distinct subset showed markedly lower scores, indicating closer proximity to the predefined ideotype. Based on the MGIDI ranking, seventeen germplasm were identified as elite and prioritized for further breeding and utilization, namely IOPPV000023, IOPPV000126, IOPPV000453, IOPPV000127, IOPPV000388, IOPPV000184, IOPPV000072, IOPPV000494, IOPPV000182, IOPPV000160, IOPPV000003, IOPPV000379, IOPPV000385, IOPPV000459, IOPPV000492.1, IOPPV000008, and IOPPV000402. This germplasm combined desirable fatty acid composition with improved oil quality, carotene content, and yield performance.

The application of MGIDI addresses an important challenge in oil palm breeding by enabling simultaneous multi-trait selection while minimizing the influence of multicollinearity among correlated variables (Sitepu et al., 2024). By estimating the Euclidean distance between individual germplasm and a predefined ideotype, MGIDI facilitates the identification of germplasm with relatively balanced performance across nutritional, physicochemical, and agronomic traits (Azrai et al., 2023). The 17 top-ranked germplasm identified in the present study, including IOPPV000023 and IOPPV000126, represent germplasm with the closest proximity to the defined multi-trait ideotype based on the integrated selection framework (Barcelos et al., 2015).

Previous studies in oil palm have reported moderate to high heritability for major compositional traits such as fatty acid profile and IV, indicating that these characteristics are influenced substantially by genetic factors (Noh et al., 2002). While heritability was not directly estimated in the present study, the integration of these traits into the MGIDI framework provides a practical basis for prioritizing germplasm for subsequent breeding evaluation (Sujadi et al., 2018). The utility of MGIDI lies in its capacity to simplify complex germplasm evaluation by integrating multiple correlated parameters into a single interpretable ranking metric, thereby supporting more systematic decision-making in breeding programs (Raj et al., 2024). This integrated chemometric approach provides a useful framework for identifying priority oil palm germplasm combining desirable lipid nutritional characteristics, carotene enrichment potential, and agronomic performance.

### 3.6 Post-selection characterization of mgidi-prioritized germplasm

**Figure 7:**
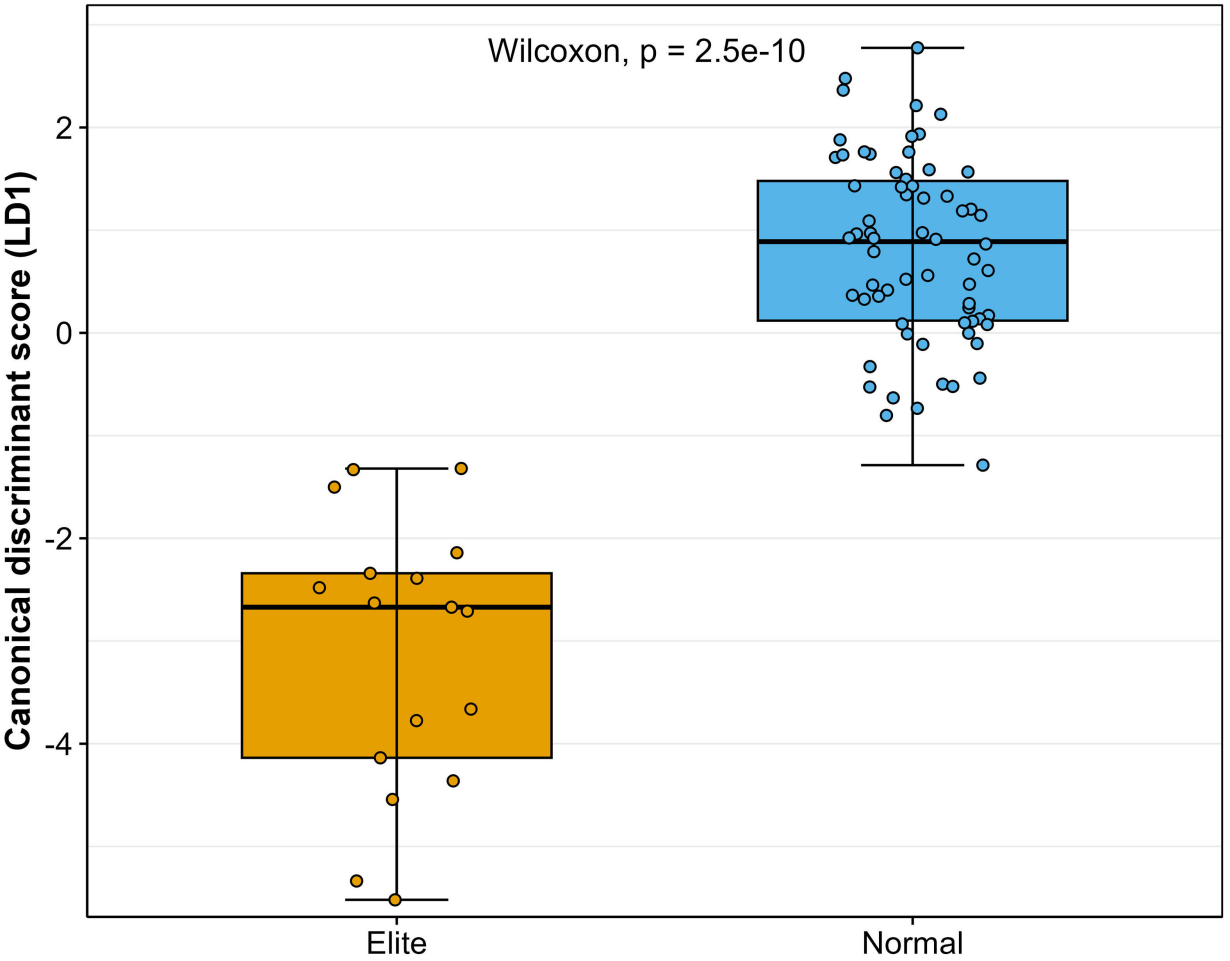
Linear discriminant analysis (LDA) showing multivariate differentiation between MGIDI-prioritized germplasm and the remaining germplasm based on the revised non-redundant trait set. The separation along the primary canonical axis illustrates differences in integrated compositional, nutritional, and agronomic characteristics between the two groups (Wilcoxon test, *p* = 1.2 × 10^-7^).

**Figure 8:**
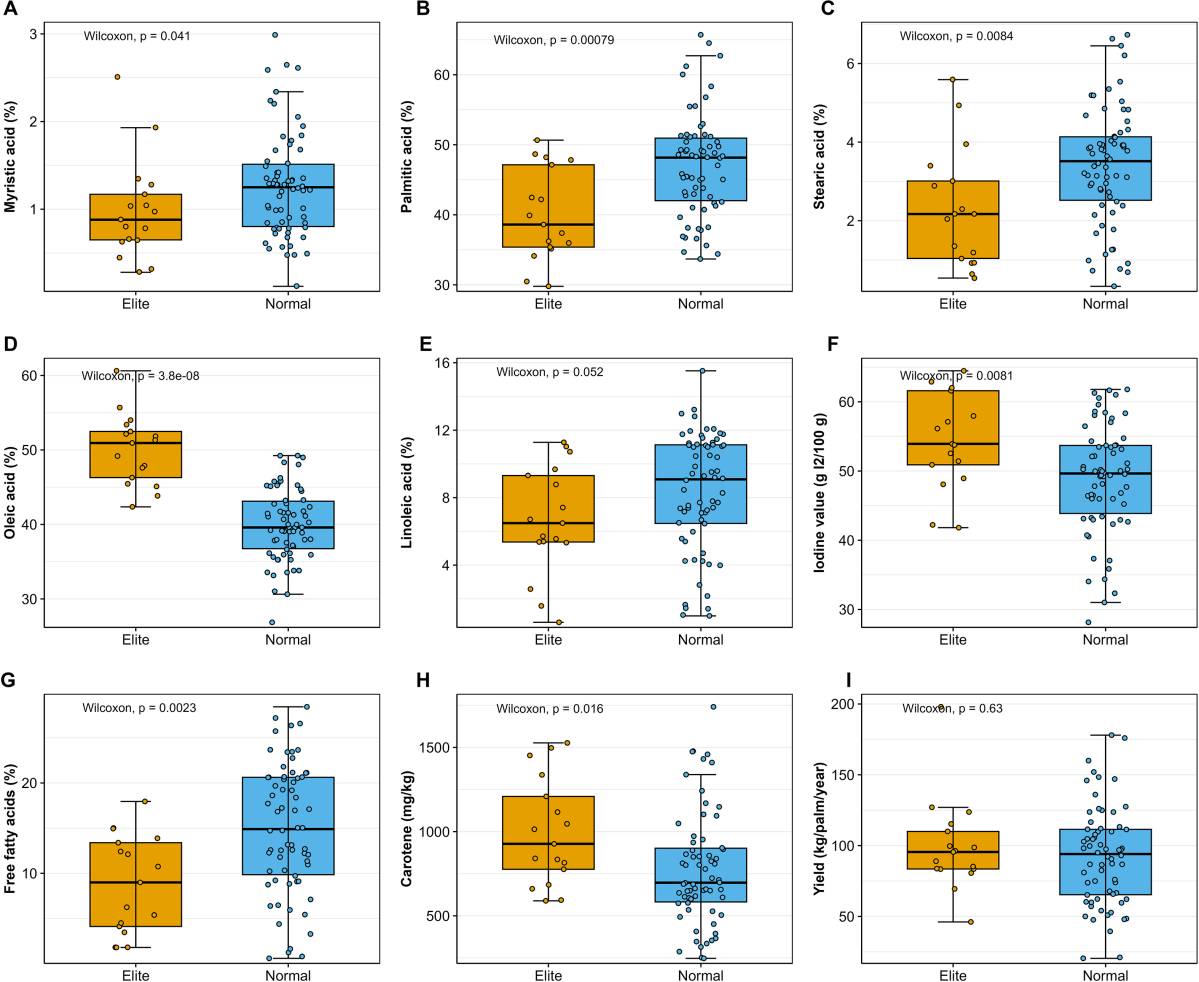
Trait-wise comparison between MGIDI-prioritized and remaining germplasm. Boxplots represent variation in **(A)** myristic acid, **(B)** palmitic acid, **(C)** stearic acid, **(D)** oleic acid, **(E)** linoleic acid, **(F)** iodine value, **(G)** free fatty acid content, **(H)** carotene concentration, and **(I)** fresh fruit bunch yield. Statistical significance was assessed using Wilcoxon rank-sum tests, and p-values are indicated above each trait.

To characterize multivariate differences between MGIDI-prioritized germplasm and the remaining germplasm, multivariate analysis of variance (MANOVA) followed by linear discriminant analysis (LDA) was performed using the same trait set employed in the MGIDI analysis (**Figures 7**, **8**). These analyses were used to describe and visualize multivariate differentiation between groups and should not be interpreted as independent validation of the MGIDI selection procedure. MANOVA revealed highly significant overall multivariate separation between the prioritized and remaining germplasm groups (Wilks’ Lambda = 0.2816, F = 13.54, df = 13, 69, p = 2.89 × 10^-^¹^4^), indicating substantial differences in their integrated compositional, nutritional, and agronomic characteristics.

Consistent with this observation, LDA demonstrated clear discrimination between prioritized and non-prioritized germplasm along the primary canonical axis (**Figure 7**). Trait-wise comparisons further highlighted the compositional features contributing to group differentiation (**Figure 8**). The prioritized germplasm exhibited significantly lower myristic acid content (p = 0.0405) and significantly higher carotene content (p = 0.0153) than the remaining germplasm. Descriptive comparisons further indicated lower palmitic acid, stearic acid, linoleic acid, AI, TI, and FFA content, together with higher oleic acid, HPI, iodine value, and fresh fruit bunch yield in the prioritized group. Although linoleic acid showed a marginal difference between groups (p = 0.0509), yield remained comparable between prioritized and non-prioritized germplasm. Overall, these results demonstrate that the revised MGIDI framework effectively enriched for germplasm combining a healthier fatty acid composition, reduced free fatty acid content, enhanced carotene concentration, and competitive yield performance within the evaluated population.

The significant multivariate differentiation identified through MANOVA and LDA supports the ability of the MGIDI-based framework to distinguish prioritized germplasm with distinct integrated compositional profiles (Raj et al., 2024). The observed contrast in lipid composition, characterized by lower saturated myristic and palmitic acid levels together with higher oleic acid content and IV, indicates that the prioritized subgroup is enriched for germplasm with comparatively more unsaturated and nutritionally favorable lipid profiles (Montoya et al., 2014). Because palmitic acid contributes strongly to the AI, the reduction in this fatty acid is consistent with improved derived nutritional lipid quality metrics (Chen and Liu, 2020). Likewise, the lower FFA content observed in the prioritized group suggests potential advantages in hydrolytic stability and crude oil quality (Yaakub et al., 2020).

An important observation from the group comparison was that carotene content was significantly higher in the prioritized germplasm, whereas fresh fruit bunch yield remained statistically comparable between prioritized and remaining germplasm groups. These findings suggest that improvement in lipid compositional quality can be achieved while simultaneously maintaining agronomic performance and enhancing carotene concentration within the evaluated population. While non-significant differences should not be interpreted as strict independence, these results suggest that improvement in lipid compositional quality may be achievable without clear adverse shifts in these traits within the evaluated population (Singh et al., 2009). This interpretation is consistent with previous studies reporting that oil composition and agronomic performance traits may exhibit partially independent patterns of phenotypic variation (Pedapati et al., 2021). Previous studies have reported opportunities for simultaneous improvement of oil quality and agronomic performance traits, supporting the practical utility of multi-trait selection approaches in oil palm breeding (Ting et al., 2016). These multivariate analyses indicate that the MGIDI-based prioritization framework effectively identified a statistically differentiated subgroup characterized by favorable integrated lipid quality traits while maintaining comparable carotene content and yield within the present dataset.

## 4 Conclusion

This study revealed substantial phenotypic diversity within African dura oil palm germplasm for fatty acid composition, lipid quality attributes, carotene content, free fatty acid content, iodine value, and fresh fruit bunch yield. The wide variation observed for key compositional traits, particularly palmitic and oleic acids, demonstrates the existence of valuable natural diversity that can be exploited in oil palm improvement programs. Multivariate analyses showed that germplasm differentiation was largely governed by the balance between saturated and unsaturated fatty acid fractions, while carotene content and yield contributed additional independent sources of variation. The integration of compositional, nutritional, and agronomic traits through a revised non-redundant Multi-Trait Genotype–Ideotype Distance Index (MGIDI) framework enabled the identification of seventeen elite germplasm (IOPPV000023, IOPPV000126, IOPPV000453, IOPPV000127, IOPPV000388, IOPPV000184, IOPPV000072, IOPPV000494, IOPPV000182, IOPPV000160, IOPPV000003, IOPPV000379, IOPPV000385, IOPPV000459, IOPPV000492, IOPPV000008, and IOPPV000402) exhibiting favorable combinations of fatty acid composition, reduced free fatty acid content, enhanced carotene concentration, and competitive yield performance. Post-selection multivariate characterization further demonstrated that these prioritized germplasms possess a distinct compositional profile compared with the remaining germplasm population. These germplasms constitute valuable donor parents for breeding programs aimed at improving oil quality while maintaining agronomic performance. Overall, the study provides a robust multi-trait framework for germplasm characterization, prioritization, and parent selection in oil palm breeding. The identified elite germplasm represents important germplasm resources for developing cultivars with improved nutritional quality, while the analytical approach presented here can facilitate evidence-based breeding decisions and accelerate genetic improvement for healthier palm oil production. Although the present study was based on phenotypic characterization, future integration of molecular markers, quantitative genetic analyses, and multi-environment validation will be necessary to confirm the underlying factors and breeding value of the prioritized germplasm.

## Data Availability

The original contributions presented in the study are included in the article/supplementary material. Further inquiries can be directed to the corresponding authors.
